# Performance of machine learning algorithms for glioma segmentation of brain MRI: a systematic literature review and meta-analysis

**DOI:** 10.1007/s00330-021-08035-0

**Published:** 2021-05-21

**Authors:** Evi J. van Kempen, Max Post, Manoj Mannil, Richard L. Witkam, Mark ter Laan, Ajay Patel, Frederick J. A. Meijer, Dylan Henssen

**Affiliations:** 1 Department of Medical Imaging, Radboud University Medical Center, Geert Grooteplein Zuid 10, 6525 EZ, Nijmegen, The Netherlands; 2 Clinic of Radiology, University Hospital Münster, Münster, Germany; 3 Department of Anaesthesiology, Pain and Palliative Medicine, Radboud University Medical Center, Nijmegen, The Netherlands; 4 Department of Neurosurgery, Radboud University Medical Center, Nijmegen, The Netherlands

**Keywords:** Machine learning, Glioma, Neuroimaging, Meta-analysis

## Abstract

**Objectives:**

Different machine learning algorithms (MLAs) for automated segmentation of gliomas have been reported in the literature. Automated segmentation of different tumor characteristics can be of added value for the diagnostic work-up and treatment planning. The purpose of this study was to provide an overview and meta-analysis of different MLA methods.

**Methods:**

A systematic literature review and meta-analysis was performed on the eligible studies describing the segmentation of gliomas. Meta-analysis of the performance was conducted on the reported dice similarity coefficient (DSC) score of both the aggregated results as two subgroups (i.e., high-grade and low-grade gliomas). This study was registered in PROSPERO prior to initiation (CRD42020191033).

**Results:**

After the literature search (*n* = 734), 42 studies were included in the systematic literature review. Ten studies were eligible for inclusion in the meta-analysis. Overall, the MLAs from the included studies showed an overall DSC score of 0.84 (95% CI: 0.82–0.86). In addition, a DSC score of 0.83 (95% CI: 0.80–0.87) and 0.82 (95% CI: 0.78–0.87) was observed for the automated glioma segmentation of the high-grade and low-grade gliomas, respectively. However, heterogeneity was considerably high between included studies, and publication bias was observed.

**Conclusion:**

MLAs facilitating automated segmentation of gliomas show good accuracy, which is promising for future implementation in neuroradiology. However, before actual implementation, a few hurdles are yet to be overcome. It is crucial that quality guidelines are followed when reporting on MLAs, which includes validation on an external test set.

**Key Points:**

*• MLAs from the included studies showed an overall DSC score of 0.84 (95% CI: 0.82–0.86), indicating a good performance.*

*• MLA performance was comparable when comparing the segmentation results of the high-grade gliomas and the low-grade gliomas.*

*• For future studies using MLAs, it is crucial that quality guidelines are followed when reporting on MLAs, which includes validation on an external test set.*

**Supplementary Information:**

The online version contains supplementary material available at 10.1007/s00330-021-08035-0.

## Introduction

Gliomas are the most frequently occurring primary tumor of the brain [1]. Accurate segmentation of gliomas on clinical magnetic resonance imaging (MRI) scans plays an important role in the quantification and objectivation of diagnosis, treatment decision, and prognosis [2–4]. In current clinical practice, T1-weighted, post-contrast T1-weighted, T2-weighted, and T2-fluid attenuated inversion recovery (FLAIR) sequences are required to characterize the different components and to assess the infiltration of the surrounding brain parenchyma [5, 6]. Glioma segmentation requires the distinguishing of tumor tissue from healthy surrounding tissues by the radiologist [7] and the segmented region of interest or volume of interest can be used to compute feature-based radiomics and quantifiable measurements [8, 9]. However, segmentation is a time-consuming task with high inter-observer variability [10, 11]. Therefore, automatic segmentation methods have been searched for as these could facilitate consistent measures and simultaneously could reduce time spent on the task by radiologists in their daily practice. These developments have been powered by the organization of the annual multimodal Brain Tumor Segmentation (BraTS) challenge (http://braintumorsegmentation.org/). Within the BraTS challenges, the organization committee released multimodal scan volumes of a relatively large number of patients suffering from glioma after which different research groups aim to construct machine learning algorithms (MLAs) to automatically segment the gliomas. The BraTS data were accompanied by corresponding segmentations which served as the ground truth [11]. Recent developments in automatic segmentation by the use of MLAs helped to achieve higher precision [12]. Within the BraTS challenges, the MLAs which yielded the most accurate results included different 2D and 3D convolutional neural networks (CNNs) [13–17], including 3D U-Nets [18, 19].

Despite the large body of scientific literature covering this topic, a comprehensive overview and meta-analysis of the accuracy of MLAs in glioma segmentation is still lacking [20, 21]. Therefore, factors which enable the further development of MLAs for glioma segmentation remain partially elusive. The aim of the current study therefore was to provide a systematic review and meta-analysis of the accuracy of MLA-based glioma segmentation tools on multimodal MRI volumes. By providing this overview, the strengths and limitations of this field of research were highlighted and recommendations for future research were made.

## Methods

The systematic review and meta-analysis was conducted in accordance with the Preferred Reporting Items for Systematic Reviews and Meta-Analyses (PRISMA) statement [22]. Prior to initiation of the research, the study protocol was registered in the international open-access Prospective Register of Systematic Reviews (PROSPERO) under number CRD42020191033.

Papers that developed or validated MLAs for the segmentation of gliomas were reviewed. Literature was searched for in MEDLINE (accessed through PubMed), Embase, and The Cochrane Library, between April 1, 2020, and June 19, 2020. No language restrictions were applied. The full search strings, including keywords and restrictions, are available in the Appendix. Studies describing MLA-based segmentation methodologies on MR images in glioma patients were included. Additional predefined inclusion criteria were as follows: (1) mean results were defined as dice similarity coefficient (DSC) score; (2) study results needed to be validated either internally and/or externally. Letters, preprints, scientific reports, and narrative reviews were included. Studies based on animals or non-human samples or that presented non-original data were excluded.

Two researchers screened the papers on title, abstract, and full-text independently. Discussions between both researchers were held to resolve all disagreements about non-consensus papers. The investigators independently extracted valuable data of the included papers using a predefined data extraction sheet after which the data was cross-checked. Data extracted from the included studies comprised the following: (a) first author and year of publication; (b) size of training set; (c) mean age of participants in the training set; (d) gender of participants in the training set; (e) size of internal test set; (f) whether there was an external validation; (g) study design, including the used MRI sequences and the segmentations which formed the ground truth; (h) architecture of the AI-algorithm(s); (i) target condition; (j) performance of the algorithm(s) in terms of DSC score, sensitivity, and specificity for both the training and the internal and/or external test sets. When studies performed external validation of the described AI-system(s), externally validated data were included in data extraction tables. Data from the internal validation were used when studies solely carried out the internal validation of the reported MLAs.

The quality of the included studies was not formally assessed, as a formal quality assessment is a well-known challenge in this area of research [23–25]. Nevertheless, Collins and Moons (2019) announced their initiative to develop a version of the transparent reporting of a multivariable prediction model for individual prognosis or diagnosis (TRIPOD) statement tailored to machine learning methods [26]. Pinto dos Santos suggested on the European Society of Radiology website various items to take into consideration when reviewing literature regarding machine learning [27]. These items were included in this review.

### Statistical assessment

An independent statistician was consulted to discuss the statistical analyses and approaches with regard to the meta-analysis. To estimate the overall accuracy of the current MLAs, a random effects model meta-analysis was conducted. To be included in the meta-analysis, studies needed to have reported the outcome of interest (i.e., DSC score), in combination with a standard deviation (SD), standard error (SE), and/or the 95% confidence interval (95% CI). For studies reporting the SE and/or the 95% CI, the SD was statistically assessed [28]. Meta-analysis was performed on aggregated data of all studies providing suitable outcomes. Then, subgroup analyses were conducted on two separate target conditions, for studies describing the segmentation of either HGGs or LGGs.

Statistical analyses were carried out by use of IBM SPSS Statistics (*IBM Corp. Released 2017. IBM SPSS Statistics for Windows, Version 25.0. IBM Corp.).* Variables and outcomes of the statistical assessment were presented as mean with ± SD when normally distributed. When data were not normally distributed, they were presented as the median with range (minimum–maximum). Statistical tests were two-sided and significance was assumed when *p* < 0.05.

The DSC score represents an overlap index and is the most used metric in validating segmentation images. In addition to the direct comparison between automated and ground truth segmentations, the DSC score is a common measure of reproducibility [29, 30]. The DSC score ranges from 0.0 (no overlap) to 1.0 (complete overlap). In this meta-analysis, a DSC score of ≥ 0.8 was considered good overlap. A DSC score of ≤ 0.5 was considered poor.

The quantitative meta-analysis was partially carried out using OpenMeta[Analyst] software, which is the visual front-end for the R package (www.r-project.org*; Metafor*) [31]. Forest plots were created to depict the estimated DSC scores from the included studies, along with the overall DSC score performance. When the 95% CI of the different subgroup analyses overlapped, no further statistical analysis was carried out.

The heterogeneity of the included studies was tested with the Higgins *I*^2^-test. The Higgins *I*^2^-test quantifies inconsistency between included studies, where a value > 75% indicates considerable heterogeneity between groups. A low heterogeneity corresponds with a Higgins *I*^*2*^ between 0 and 40% [28]. Both the meta-analyses of the aggregated groups as the meta-analyses of the subgroups were performed using a random effects model, due to an observed high heterogeneity (Higgins *I*^2^ > 75%) between included studies [32].

To showcase possible publication bias, a funnel plot was created by means of Stata (*StataCorp. 2019. Stata Statistical Software: Release 16.: StataCorp LLC.).*

## Results

**Fig. 1:**
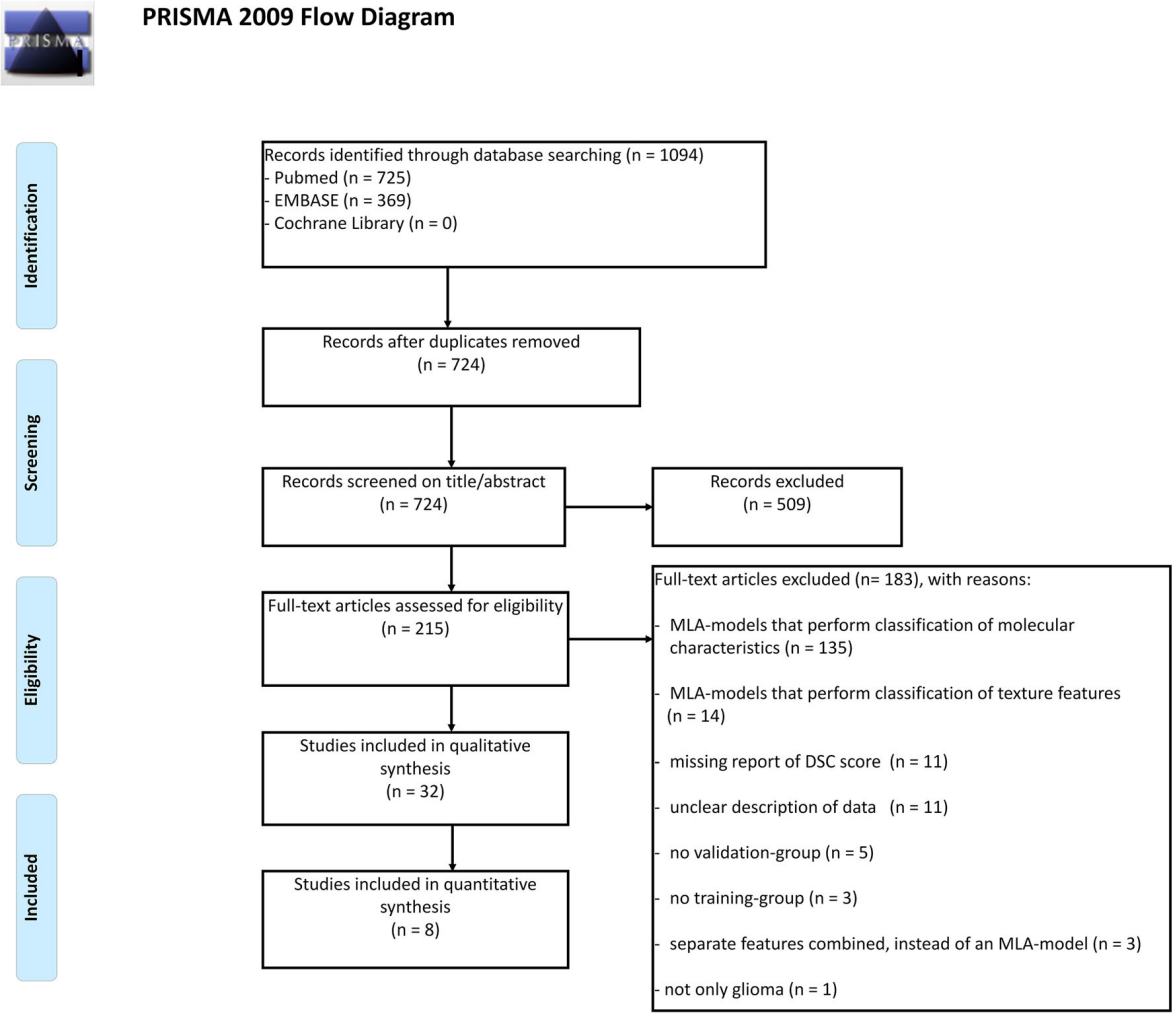
PRISMA flowchart of systematic literature search

Initially, 1094 publications were retrieved through database searching. An additional ten publications were identified through cross-referencing. After removing duplicates, the remaining 734 publications were screened. Based on the title and abstract, 509 papers were excluded. A total of 225 full-text articles were assessed for eligibility and 42 studies were included in the systematic review. Ten studies were eligible for inclusion for the meta-analysis as they provided sufficient quantitative data (e.g., only these studies provided the DSC score along with SD for the performance of the MLA) (Fig. 1). Publications describing the use of (automated) segmentations to apply MLAs to classify molecular characteristics of gliomas (*n* = 135) were excluded. Fourteen papers were excluded as they described the use of MLAs on gliomas to perform texture analyses. Eleven papers did not report the DSC score and another 11 studies showed unclarities in data reporting. Contacting the authors of these papers did not result in the acquisition of the needed data. Five studies did not report results of internal or external validation steps, whereas an additional three studies did not report data from the training-group. Three studies described separate combined features, instead of a coherent MLA methodology. One study was excluded due to the inclusion of other brain tumors next to gliomas (e.g., metastases) (Fig. 1).

### Review of the included studies

Based on the full-text analysis, 42 segmentation studies [13, 17, 33–72] were included for the systematic review, from which the participant demographics and study characteristics are depicted in Table 1. The used MLAs are presented in Table 1 and comprised different types of CNNs [13, 17, 34, 35, 37–43, 45–47, 49–53, 55–57, 60, 61, 63–65, 67] and random forest model [68–70], multiple classifier system [33, 44], and an adaptive superpixel generation algorithm [60]. In addition, one study used semi-automatic constrained Markov random field pixel labeling [64], one study used an end-to-end adversarial neural network [71], and one study used a 3D supervoxel-based learning method [56].

**Table 1:** Participant demographics, study characteristics, and outcomes of the included studies and performance evaluation of MLAs of the included studies

	Training set			Test set					Reference segmentations							
First author (year of publication) (reference)	N	Mean age (years)	M-F	N	External|validation	Target condition	Dataset	MR sequences		Summary of DLA methods	2D vs. 3D	Subgroups	SN	SP	DSC score (± SD)	Data/code openly available?
Kamnitsas et al (2017) [17]	274	NR	NR	110	No	HGG and LGG	BraTS 2015	T1w, T2w, T1w c+, and FLAIR images	BraTS segmentations	3D CNN with two-scale extracted Features and 3D dense Conditional Random Field as postprocessing	3D	Whole tumor	88	NR	0.85	Y/Y
												Contrast enhancing tumor	67	NR	0.63	
												Tumor core	60	NR	0.67	
*Amirmoezzi et al (2019)* [33]	80	NR	NR	80	No	HGG and LGG	BraTS 2012	FLAIR images	BraTS segmentations	A specific region of interest (ROI) that contains tumor was identified and then the intensity non-uniformity in ROI was corrected via the histogram normalization and intensity scaling. Each voxel in ROI was presented using 22 features and then was categorized as tumor or non-tumor by a multiple classifier system	3D	Simulated data	84.0	98.0	0.81 ± 0.10	Y/N
												Real data	89.0	98.0	0.80 ± 0.10	
Banerjee et al (2020) [34]	285	NR	NR	66	No	HGG and LGG	BraTS 2018	T1w, T2w, T1w c+, and FLAIR images	BraTS segmentations	Encoder-decoder type CNN model combined with a consensus fusion strategy with a fully connected Conditional random field-based post-refinement	3D	Whole tumor	91.4	99.3	0.902	Y/Y
												Contrast enhancing tumor	86.9	99.7	0.824	
												Central tumor necrosis	87.4	99.7	0.872	
Bonte et al (2018) [35]	287	NR	NR	285	Yes	HGG, LGG, and other tumor types (e.g., meningioma, ependymoma )	BraTS 2013, BraTS 2017, and original data	T1w c+, and FLAIR images	BraTS segmentations	Random Forests model combining voxel-wise texture and abnormality features on 275 feature maps	3D	LGG – whole tumor	NR	NR	0.684	Y/N
												LGG – tumor core	NR	NR	0.409	
												HGG – whole tumor	NR	NR	0.801	
												HGG- tumor core	NR	NR	0.750	
*Choi et al (2020*) [36]	45	58.7	24–21	46	Yes	HGG	Original data, TCIA data, and TCGA data	T2w images	Manual segmentations made by two experienced radiologists	V-Net model using 3D input and output which uses convolution with a stride of factor 2 instead of max-pooling	3D	Tumor + peritumoral edema	NR	NR	0.78 ± 0.14	Y/Y
Cui et al (2018) [37]	240	NR	NR	34	No	HGG and LGG	BraTS 2015	T1w, T2w, T1w c+, and FLAIR images	BraTS segmentations	Fully convolutional network in conjunction with the transfer learning technology combined with a CNN with deeper architecture and smaller kernel to label a defined tumor region into multiple subregions	2D	Whole tumor	NR	NR	0.89	Y/N
Hasan et al (2018) [38]	285	NR	NR	146	No	HGG and LGG	BraTS 2017 and original data	T1w, T2w, T1w c+, and FLAIR images	BraTS segmentations	Nearest neighbor re-sampling based elastic-transformed U-net deep CNN framework	2D	HGG	NR	NR	0.899	Y/N
												LGG	NR	NR	0.846	
												Combined	NR	NR	0.872	
Havaei et al (2017) [39]	30	NR	NR	10	No	HGG and LGG	BraTS 2013	T1w, T2w, T1w c+, and FLAIR images	BraTS segmentations	A CNN with two pathways of both local and global information	3D	Whole tumor	84	88	0.840	Y/N
												Contrast enhancing tumor	68	54	0.570	
												Tumor core	72	79	0.710	Y/N
Havaei et al (2016) [40]	30	NR	NR	10	No	HGG and LGG	BraTS 2013	T2w, T1w c+, and FLAIR images	BraTS segmentations	A cascade neural network architecture in which the output of a basic CNN is treated as an additional source of information for a subsequent CNN	3D	PKSVM-CRF	78	88	0.86	
												KSVM-CRF	82	87	0.84	
												kNN-CRF	78	91	0.85	
Hussain et al (2017) [41]	30	NR	NR	NR	No	HGG and LGG	BraTS 2013	T1w, T2w, T1w c+, and FLAIR images	BraTS segmentations	deep cascaded convolutional neural networks	2D	Whole tumor	82	85	0.80	Y/N
												Contrast enhancing tumor	57	60	0.57	
												Tumor core	63	82	0.67	
Iqbal et al (2019) [42]	274	NR	NR	110	No	HGG and LGG	BraTS 2015	T1w, T2w, T1w c+, and FLAIR images	BraTS segmentations	Combination of CNN- and long short-term memory models	2D	Whole tumor	NR	NR	0.823	Y/N
Iqbal et al (2018) [43]	274	NR	NR	110	No	HGG and LGG	BraTS 2015	T1w, T2w, T1w c+, and FLAIR images	BraTS segmentations	CNN Model	2D	SkipNet**	83	73	0.87	Y/N
												SENet**	86	83	0.88	
												IntNet**	86	73	0.90	
*Jiang et al (2013)* [44]	80	NR	NR	23	No	HGG and LGG	BraTS 2012	T1w, T2w, T1w c+, and FLAIR images	BraTS segmentations	Method exploiting the global classifier (trained by using samples from the population feature set) and a custom classifier (trained by using samples from seed points in the testing image). The outputs of these two classifiers are weighted and then constructed	3D	Whole tumor	87.2	83.1	0.845 ± 0.09	Y/N
Kao et al (2019) [45]	285	NR	NR	66	No	HGG and LGG	BraTS 2017 and BraTS 2018	T1w, T2w, T1w c+, and FLAIR images	BraTS segmentations	3D CNN with two-scale extracted features and 3D dense conditional random field as postprocessing combined with a separate 3D U-Net	3D	Whole tumor	NR	NR	0.908	Y/N
												Contrast enhancing tumor	NR	NR	0.782	
												Tumor core	NR	NR	0.823	
Li et al (2017) [46]	59	NR	NR	101	No	LGG	Original data	FLAIR images	Manual segmentations made by two experienced neurosurgeons	3D CNN with two-scale extracted features and 3d dense conditional random field as postprocessing	3D	Whole tumor	88.9	NR	0.802	N/N
*Liu et al (2018)* [47]	200	NR	NR	74	No	HGG and LGG	BraTS 2015	T1w, T2w, and FLAIR images	BraTS segmentations	3D patch-based fully convolution network adopting the architecture of V-Net	3D	Whole tumor	NR	NR	0.87 ± 0.06	Y/N
Meng et al (2018) [48]	154	NR	NR	22	No	HGG and LGG	BraTS 2015	T1w, T2w, T1w c+, and FLAIR images	BraTS segmentations	Light noise suppression U-network to achieve end-to-end learning without elaborate pre-processing and postprocessing	2D	Whole tumor	82	74	0.89	Y/N
Naceur et al (2018) [49]	285	NR	NR	NR	No	HGG and LGG	BraTS 2017	T1w, T2w, T1w c+, and FLAIR images	BraTS segmentations	Three end-to-end incremental deep convolutional neural network models	2D	Whole tumor	82	74	0.89	Y/N
Naser et al (2020) [50]	110	46	54-56	110	No	LGG	TCIA	T1w, T1w c+, and FLAIR images	Manual segmentations made by the investigators	A deep learning approach which combines CNNs based on the U-net for tumor segmentation and transfer learning based on a pre-trained convolution-base of Vgg16 and a fully connected classifier for tumor grading was developed.	3D	Whole tumor	NR	NR	0.84	Y/N
*Perkuhn et al (2018*) [51]	*	NR	NR	64	Yes	HGG	Original data	T1w, T2w, T1w c+, and FLAIR images	Manual segmentations made by the investigators following the BraTS challenge workflow	3D CNN with two-scale extracted features and 3D dense Conditional random Field as postprocessing	3D	Whole tumor	84	NR	0.86 ± 0.09	Y/N
												Contrast enhancing tumor	78	NR	0.78 ± 0.15	
												Central tumor necrosis	57	NR	0.62 ± 0.30	
Razzak et al (2019) [52]	285	NR	NR	110	No	HGG and LGG	BraTS 2013 and BraTS 2015	T1w, T2w, T1w c+, and FLAIR images	BraTS segmentations	Two-pathway CNN which simultaneously accommodates the global and local features as well as embedding additional transformations like rotations and reflections in itself by applying not only translation but also rotational and reflection to the filters which result in an increase in the degree of weight sharing	2D	Whole tumor	88.3	NR	0.892	Y/N
Savareh et al (2019) [53]	274	NR	NR	NR	No	HGG and LGG	BraTS 2015	T1w, T2w, T1w c+, and FLAIR images	BraTS segmentations	Fully convolutional network was selected to implement the wavelet-enhanced fully convolutional network model	3D	Whole tumor	93	99	0.918	Y/N
*Soltaninejad et al (2018)* [54]	11	53	NR	11	No	HGG and LGG	BraTS 2013 and original data	T1w, T2w, T1w c+, FLAIR, and DTI images	Segmentations derived from the BraTS dataset combined with manual segmentations made by the investigators following the BraTS challenge workflow	3D supervoxel-based learning method. Supervoxels are generated using the information across the multimodal MRI dataset. For each supervoxel, a variety of features including histograms of tex-ton descriptor, calculated using a set of Gabor filters with different sizes and orientations, and first-order intensity statistical features are extracted. Those features are fed into a random forests classifier to classify each supervoxel into tumor core, edema, or healthy brain tissue.	3D	Whole tumor	NR	NR	0.84 ± 0.06	Y/N
Sun et al (2019) [55]	274	NR	NR	110	No	HGG and LGG	BraTS 2015	T1w, T2w, T1w c+, and FLAIR images	BraTS segmentations	3D CNN-based method	3D	Whole tumor	89	NR	0.84	Y/N
												Contrast enhancing tumor	69	NR	0.62	
Wang et al (2018) [56]	100	NR	NR	NR	No	HGG and LGG	NR	NR	NR	3D-CNN Model	3D	Whole tumor	NR	NR	0.916	N/N
Wu et al (2020) [57]	285	NR	NR	66	No	HGG and LGG	BraTS 2017	T1w, T2w, T1w c+, and FLAIR images	BraTS segmentations	2D U-Nets	2D	Whole tumor	NR	NR	0.91	Y/Y
												Contrast enhancing tumor	NR	NR	0.80	
												Tumor core	NR	NR	0.83	
*Wu et al (2019)* [58]	228	NR	NR	57	No	HGG and LGG	BraTS 2017	T2w images	BraTS segmentations	An adaptive superpixel generation algorithm based on simple linear iterative clustering version with 0 parameter (ASLIC0) was used to acquire a superpixel image with fewer superpixels and better fit the boundary of ROI by automatically selecting the optimal number of superpixels.	2D	Whole tumor	81.5	99.6	0.849 ± 0.07	Y/N
Yang et al (2019) [59]	255	NR	NR	30	No	HGG and LGG	BraTS 2017	T1w, T2w, T1w c+, and FLAIR images	BraTS segmentations	U-net	2D	Whole tumor	90.6	NR	0.883 ± 0.06	Y/N
												Contrast enhancing tumor	79.2	NR	0.784 ± 0.10	
												Tumor core	88.3	NR	0.781 ± 0.10	
Yang et al (2019) [60]	274	NR	NR	274	No	HGG and LGG	BraTS 2015	T1w, T2w, T1w c+, and FLAIR images	BraTS segmentations	Two-pathway convolutional neural network combined with random forests	2D	SK-TPCNN – Whole tumor	95	NR	0.86	Y/N
												SK-TPCNN – contrast-enhancing tumor	76	NR	0.81	
												SK-TPCNN – tumor core	91	NR	0.74	
												SK-TPCNN + RF – whole tumor	96	NR	0.89	
												SK-TPCNN + RF – contrast-enhancing tumor	83	NR	0.87	
												SK-TPCNN + RF – Tumor core	92	NR	0.80	
Yang et al (2020) [61]	274	NR	NR	274	No	HGG and LGG	BraTS 2015	T1w, T2w, T1w c+, and FLAIR images	BraTS segmentations	2D-CNN Model	2D	Whole tumor	88	NR	0.90	Y/N
												Contrast enhancing tumor	84	NR	0.88	
												Tumor core	82	NR	0.82	
*Zhao et al (2013)* [62]	30	NR	NR	30	No	HGG	BraTS 2012	T1w, T2w, T1w c+, and FLAIR images	BraTS segmentations	Semi-automatic Constrained Markov random field pixel labeling	3D	HGG	NR	NR	0.835 ± 0.089	Y/N
						LGG						LGG	NR	NR	0.848 ± 0.087	
Zhou et al (2020) [63]	285	NR	NR	66	No	HGG and LGG	BraTS 2013, BraTS 2015, and BraTS 2018	T1w, T2w, T1w c+, and FLAIR images	BraTS segmentations	3D dense connectivity model	3D	Whole tumor	NR	NR	0.864	Y/N
												Contrast-enhancing tumor	NR	NR	0.753	
												Tumor core	NR	NR	0.774	
Zhuge et al (2017) [64]	20	NR	NR	10	Yes	HGG	BraTS 2013 dataset and original data	T1w, T2w, T1w c+, and FLAIR images	Segmentations derived from the BraTS dataset [11]; original data was manually annotated following the BraTS-protocol [11]	Holistically nested CNN model	2D	Whole tumor	85.0	NR	0.83	Y/Y
Dong et al (2017) [65]	274	NR	NR	NR	No	HGG	BraTS 2015	T1w, T2w, T1w c+, and FLAIR images	BraTS segmentations	U-Net based deep convolutional networks	3D	LGG - whole tumor	NR	NR	0.84	Y/N
												LGG – tumor core	NR	NR	0.85	
												HGG – whole tumor	NR	NR	0.88	
												HGG – contrast-enhancing tumor	NR	NR	0.81	
												HGG – tumor core	NR	NR	0.87	
*Dvorak and Menze (2015)* [66]	163	NR	NR	25	No	HGG and LGG	BraTS 2013	T1w, T2w, T1w c+, and FLAIR images	BraTS segmentations	Structured prediction was used together with a CNN	3D	LGG - whole tumor	NR	NR	0.85 ± 0.06	Y/N
												LGG – tumor core	NR	NR	0.65 ± 0.15	
												HGG – whole tumor	NR	NR	0.80 ± 0.17	
												HGG – contrast-enhancing tumor	NR	NR	0.81 ± 0.11	
												HGG – tumor core	NR	NR	0.85 ± 0.08	
Lyksborg et al (2015) [67]	91	NR	NR	40	No	HGG and LGG	BraTS 2014	T1w, T2w, T1w c+, and FLAIR images	BraTS segmentations	An ensemble of 2D CNNs with a three-step volumetric segmentation	2D	Whole tumor	82.5	NR	0.810	Y/N
Pereira et al (2016) [13]	30	NR	NR	NR	No	HGG and LGG	BraTS 2013	T1w, T2w, T1w c+, and FLAIR images	BraTS segmentations	A CNN with small 3 × 3 kernels	2D	Whole tumor	86	NR	0.88	Y/Y
Pinto et al (2015) [68]	40	NR	NR	10	No	HGG and LGG	BraTS 2013	T1w, T2w, T1w c+, and FLAIR images	BraTS segmentations	Using appearance- and context-based features to feed an extremely randomized forest	2D	Whole tumor	82	NR	0.83	Y/N
												Contrast-enhancing tumor	79	NR	0.73	
												Tumor core	75	NR	0.78	
Tustison et al (2015) [69]	30	NR	NR	10	No	HGG and LGG	BraTS 2013	T1w, T2w, T1w c+, and FLAIR images	BraTS segmentations	Combine a random forest model with a framework of regularized probabilistic segmentation	2D	Whole tumor	89	NR	0.87	Y/Y
												Contrast-enhancing tumor	83	NR	0.74	
												Tumor core	88	NR	0.78	
Usman and Rajpoot (2017) [70]	30	NR	NR	NR	No	HGG and LGG	BraTS 2013	T1w, T2w, T1w c+, and FLAIR images	BraTS segmentations	Automated wavelet-based features + a random forest classifier	3D	Whole tumor	NR	NR	0.88	Y/N
												Contrast-enhancing tumor	NR	NR	0.95	
												Tumor core	NR	NR	0.75	
Xue et al (2017) [71]	274	NR	NR	NR	No	HGG and LGG	BraTS 2015	T1w, T2w, T1w c+, and FLAIR images		An end-to-end adversarial neural network	2D	Whole tumor	80	NR	0.85	Y/Y
												Contrast-enhancing tumor	62	NR	0.66	
												Tumor core	65	NR	0.70	
*Zikic et al (2012)* [72]	30	NR	NR	10	No	HGG	BraTS 2012	T1w, T2w, T1w c+, and FLAIR images	BraTS segmentations	Apply a CNN in a sliding-window fashion in the 3D space	3D	Whole tumor	NR	NR	0.90 ± 0.09	Y/N
												Contrast-enhancing tumor	NR	NR	0.85 ± 0.09	
												Necrotic tumor core	NR	NR	0.75 ± 0.16	
												Peritumoral edema	NR	NR	0.80 ± 0.18	

Studies included in the meta-analysis were italicized

*BraTS*, Brain Tumor Image Segmentation Benchmark; *CNN*, convolutional neural network; *DSC*, dice similarity coefficient; *kNN-CRF*, k-nearest neighbor conditional random fields; *KSVM-CRF*, kernel support vector machine with rbf kernel conditional random fields; *LSTM*, long short-term memory; *MLA*, machine learning algorithms; *N*, no; *NR*, not reported; *PKSVM-CRF*, proposed product kernel support vector machine conditional random fields; *SD*, standard deviation; *SK-TPCNN (+RF)*, small kernels two-path convolutional (+ random forests) neural network; *SN*, sensitivity; *SP*, specificity; *TCIA*, the Cancer Imaging Archive; *TCGA*, the Cancer Genome Atlas; *Y*, yes. *The deep learning model is based on the recently published DeepMedic architecture, which provided top scoring results on the BRATS data set [17]. **Data separated by LGG and HGG for each network available in the original paper

For more information on the multivendor BraTS dataset, see Menze et al [11]. Please note that the ground truth of BraTS 2015 was first produced by algorithms and then verified by annotators; in contrast, the ground truth of BraTS 2013 fused multiple manual annotations

Thirty-eight studies combined different combinations of MRI sequences for brain tumor segmentation (Table 1) [13, 17, 33–42, 44, 45, 47–57, 59–72]. Only 3 studies used one MRI sequence for the algorithm to segment [43, 46, 58]. One conference paper did not report on the used MRI sequences [56]. Four studies reported not to have used (any part of) the BraTS datasets [36, 46, 50, 51]. Two of these papers used original data [46, 51]. The other two papers used either data from the Cancer Imaging Archive (TCIA) [50] or a combination of TCIA data and original data [36].

In 36 studies, the ground truth (i.e., segmentations) was derived from the BraTS dataset [13, 17, 33–36, 38–45, 47–49, 52–55, 57–72]. In two of these studies, the researchers added segmentations of additional original data. Segmentations were manually annotated by two experienced professionals independently following the BraTS segmentation protocol[54, 64]. In one paper, only original data with corresponding segmentations were used. These segmentations were made independently by two experienced professionals following the BraTS segmentation protocol [51]. Three papers used segmentations which were obtained without adhering to the BraTS segmentation protocol [36, 46, 50]. In one conference paper, the segmentation methodology was not described [56]. Please note that the ground truth segmentations of BraTS 2015 were first produced by algorithms and then verified by annotators, whereas the ground truth of BraTS 2013 fused multiple manual annotations.

The performance of the MLAs, in terms of sensitivity, specificity, and DSC score, is displayed in Table 1. All studies used retrospectively collected data. Nine studies focused specifically on the segmentation of HGGs, whereas seven studies focused on the segmentation of LGGs. The remaining studies (*n* = 31) described the segmentation of gliomas in general without the subdivision of LGG and HGG. Five of the included studies [33, 35, 38, 62, 65] described segmentation of multiple target conditions (i.e., segmentation of both HGG and LGG). For these studies, the results of each different target are displayed in Table 1 as well. All of the included studies conducted some version of cross-validation on the MLAs; however, only four studies [35, 36, 51, 64] performed an external validation of performance.

Nine studies [33, 35, 36, 38, 51, 62, 64, 65, 72] described the segmentation of HGGs in particular, with four studies [35, 36, 51, 64] externally validating the performance of the reported MLAs. Performance evaluation of the included studies in terms of the validated DSC score ranged from 0.78 to 0.90. MLA sensitivity ranged from 84 to 85% (*n* = 3) [33, 51, 64]. Only one study [33] presented the specificity rate (i.e., 98%).

Seven studies [33, 35, 38, 46, 50, 62, 65] described the segmentation of LGGs. External validation of the MLA was performed by one study [35]. The validated DSC score for the included studies ranged from 0.68 to 0.85. Sensitivity was 89% (*n* = 2) [33, 46], whereas specificity was 98% (*n* = 1) [33].

### Meta-analysis of the included studies

**Fig. 2:**
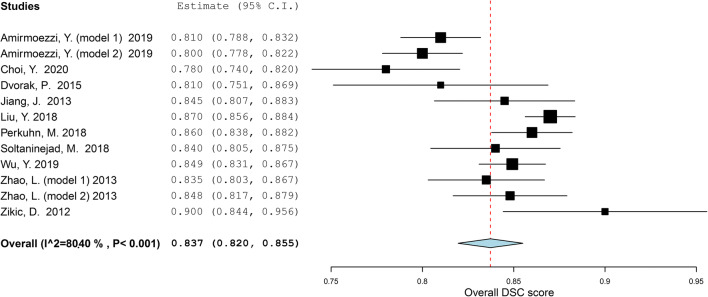
Forest plot of the included studies that assessed the accuracy of segmentation of glioma. Legend: DSC, dice similarity coefficient; CI, confidence interval. Forest plot shows that the performance of the MLAs to segment gliomas are centered around a DSC of 0.837 with a 95% CI ranging from 0.820 to 0.855

The aggregated meta-analysis comprised twelve MLAs, described in ten individual studies [33, 36, 44, 47, 51, 54, 58, 62, 66, 72], and showed an overall DSC score of 0.84 (95% CI: 0.82 – 0.86) (Fig. 2). Heterogeneity showed to be 80.4%, indicating that studies differed significantly (*p* < 0.001).

**Fig. 3:**
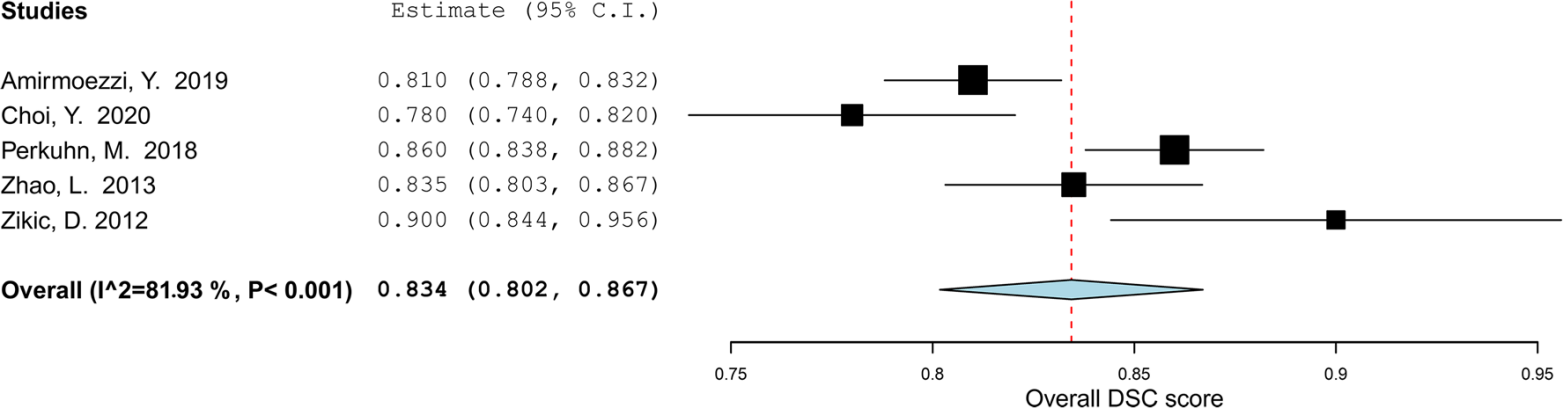
Forest plot of the included studies that assessed the accuracy of segmentation of high-grade glioma. Legend: DSC, dice similarity coefficient; CI, confidence interval. Forest plot shows that the performance of the MLAs to segment HGGs are centered around a DSC of 0.834 with a 95% CI ranging from 0.802 to 0.867

**Fig. 4:**
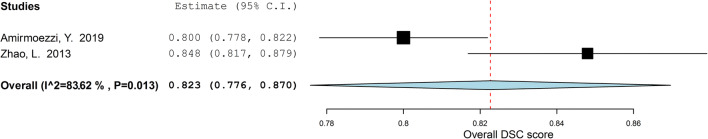
Forest plot of the included studies that assessed the accuracy of segmentation of low-grade glioma. Legend: DSC, dice similarity coefficient; CI, confidence interval. Forest plot shows that the performance of the MLAs to segment LGGs are centered around a DSC of 0.823 with a 95% CI ranging from 0.776 to 0.870

For the subgroup analysis of segmentation studies focusing on HGGs, the results are depicted in Fig. 3. Overall, DSC score for the five included studies [33, 36, 51, 62, 72] was 0.83 (95% CI: 0.80 – 0.87). The estimated *I*^2^ heterogeneity between groups showed to be 81.9% (*p = *0.001). Two studies [33, 62] focusing on the segmentation of LGGs were included in another subgroup meta-analysis. Overall, the DSC score was found to be 0.82 (95% CI: 0.78–0.87) (Fig. 4). The estimated heterogeneity of included groups was 83.62% (*p* = 0.013). Hence, the heterogeneity was determined as high for both subgroup meta-analyses.

### Publication bias

**Fig. 5:**
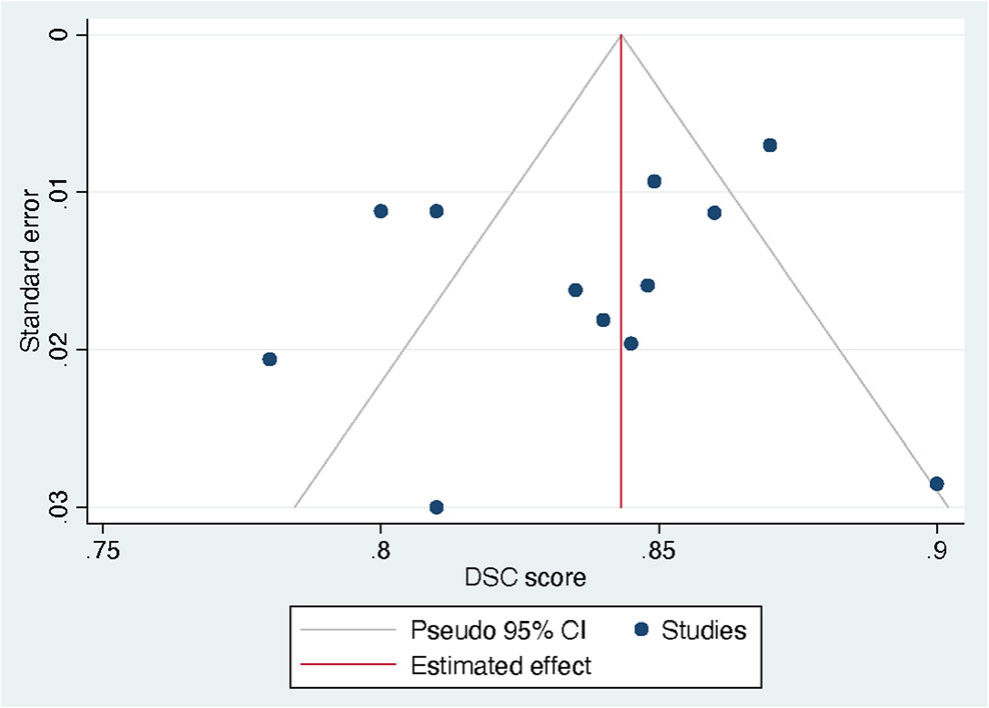
Funnel plot of the included studies. Legend: DSC, dice similarity coefficient; CI, confidence interval. DSC score was displayed on the horizontal axis as the effect size; SE was plotted on the vertical axis of the funnel plot

Studies included in the funnel plot were the ten studies that were meta-analyzed (Fig. 5). The funnel plot showed an asymmetrical shape, giving an indication for publication bias among included studies. Besides, not all studies were plotted within the area under the curve of the pseudo-95% CI, supporting the indication of possible publication bias [28].

## Discussion

Various MLAs for the automated segmentation of gliomas were reviewed. Although heterogenous, MLAs showed to have a good DSC score with no differences between the segmentation of LGG and HGG. However, there were some indications for publication bias within this field of research.

Currently, segmentation of tumor lesions is a subjective and time-consuming task [58]. By replacing the current manual methods with an automated computer-aided approach, improvement of glioma quantification and subsequently radiomics can be achieved. However, automated segmentation of gliomas is a challenging task, due to the large variety of morphological tumor characteristics among patients [11]. As HGGs usually show more heterogeneous MRI characteristics, their automated segmentation could be expected to be more challenging compared to LGGs. Furthermore, the low proliferative state of LGGs likely results in lower perfusion and higher diffusion values in affected tissue [73, 74]. No performance difference was observed between the segmentation of HGGs and LGGs. Given the differences between HGGs and LGGs, it was expected that significant differences would arise in automatic segmentation tasks. Nevertheless, the ground truth segmentations were based on manual delineation by a (neuro)radiologist, indicating that the performance of automatic segmentation could only be as good as the ground truth segmentations. In addition, the ground truth of BraTS 2015 was first produced by algorithms and then verified by annotators, whereas the ground truth of BraTS 2013 fused multiple manual annotations.

Although MLAs performing automated segmentation show quite promising results (overall DSC score of 0.84; 95% CI: 0.82–0.86), there is still no wide acceptance and implementation of these methodologies in daily clinical practice. One of the explanations for this can be found in the different MLA methodologies; different MLA approaches and their exact details have a significant impact on the outcomes, even when applied to the same dataset. For example, in the BraTS 2019 challenge, the top three with regard to the segmentation task comprised a two-stage cascaded U-Net [75], a deep convolution neural network [76], and an ensemble of 3D-to-2D CNNs [77].

Another reason may be the absence of standardized procedures on how to properly use these segmentation systems. There are substantial differences between advanced systems that offer computer-aided segmentation and the current standards for neuroradiologists, which impedes the integration of MLA methods. CE-certified software is limitedly available in clinical practice, which is one of the reasons for the impediment. Also, the purpose for the use of MLAs varies; where radiologists mainly use these techniques for follow-up, neurosurgeons mostly use MLAs for therapeutic planning. In addition, direct integration into the neuroradiologist’s daily practice without extra time spent on the task will be needed to make automatic glioma segmentation feasible. Moreover, the current automated segmentations still need to be supervised by trained observers. It seems more likely that implementation of MLAs in neuroradiology will lead to an interaction between doctor and computer so that neuroradiologists will utilize more advanced technologies in the establishment of diagnoses [78]. The future implementation of MLAs in the diagnosis of glioma is of great clinical relevance, as these algorithms can support the non-invasive analysis of tumor characteristics without the need of histopathological tissue assessment. More specifically, automatic segmentations form the basis of further sophisticated analyses to clarify meaningful and reliable associations between neuroimaging features and survival rate [79, 80]. In conclusion, as automated segmentation of glioma is considered to be the first step in this process, the implementation of MLAs holds great potential for the future of neuroradiology.

Various publications were found with regard to the automated segmentation of gliomas in the post-operative setting [81–84]. Quantitative metrics are believed to be needed for therapy guidance, risk stratification, and outcome prognostication in the post-operative setting. MLAs could also represent a potential solution for automated quantitative measurements of the burden of disease in the post-operative setting. As shown in Table 2, however, the DSC scores of these studies are lower as compared to the DSC scores of the pre-operative MLA-based segmentations [81–84]. An explanation for these differences in performance could be the post-surgical changes of the brain parenchyma and the presence of air and blood products in the post-operative setting. Together these factors have been reported to affect the performance of MLAs [81].

**Table 2:** Overview of the studies on post-operative glioma segmentation

	Training set			Test set					Reference segmentations							
First author (year of publication) (reference)	N	Mean age (years)	M-F	N	External validation	Target condition	Dataset	MR Sequences		Summary of DLA methods	2D vs. 3D	Subgroups	SN	SP	DSC score (± SD)	Data/code openly available?
Herrmann et al (2020) [81]	30	NR	NR	30	No	Brain resection cavity delineation	Original data	T1w, T2w, T1w c+, and FLAIR images	Manual segmentations made by three experienced radiation oncology experts. To improve inter-rater consistency the raters have been instructed by an experienced neuro-radiologist.	A fully convolutional densely connected architecture which builds on the idea of DenseNet was used.	3D	NA	NR	NR	0.83	N/N
Meier et al (2016) [82]	14	NR	NR	14	No	Brain volume delineation during and after therapy with neurosurgery, radiotherapy, chemotherapy, and/or anti-angiogenic therapy	Original data	T1w, T2w, T1w c+, and FLAIR images	Manual segmentations made by two raters (one experienced, one inexperienced); this table only represents the overlap between the MLA and the experienced rater	Machine learning–based framework using voxel-wise tissue classification for automated segmentation	2D	Non- enhancing T2 hyperintense tissue	NR	NR	0.673	N/N
												Contrast-enhancing T2 hyperintense tissue	NR	NR	0.183	
Zeng et al (2016) [83]	218	NR	NR	191	No	Segmenting post-operative scans	BraTS 2016 and original data	T1w, T2w, T1w c+, and FLAIR images	BraTS segmentations	A hybrid generative-discriminative model was used. Firstly, a generative model based on a joint segmentation-registration framework was used to segment the brain scans into cancerous and healthy tissues. Secondly, a gradient boosting classification scheme was used to refine tumor segmentation based on information from multiple patients.	3D	Post-operative HGG – Whole tumor	NR	NR	0.72	N/N
												Post-operative HGG – contrast-enhancing tumor	NR	NR	0.49	
												Post-operative HGG – tumor core	NR	NR	0.57	
Tang et al (2020) [84]	59	41.2 ± 12.6	32-27	15	No	Post-operative glioma segmentation in CT images	Original data	T1w, T2w, T1w c+, and FLAIR images	Manual segmentations made by one experienced radiation oncology expert	DFFM is a multi-sequence MRI–guided CNN that iteratively learned the deep features from CT images and multi-sequence MR images simultaneously by utilizing a multi-channel CNN architecture, and then combined these two deep features together to produce the segmentation result. The whole network was optimized together via a standard back-propagation.	3D	NA	NR	NR	0.818	N/N

*BraTS*, Brain Tumor Image Segmentation Benchmark; *CNN*, convolutional neural network; *DSC*, dice similarity coefficient; *MLA*, machine learning algorithms; *N*, no; *NA*, not applicable; *NR*, not reported; *SD*, standard deviation; *SN*, sensitivity; *SP*, specificity; *Y*, yes

For more information on the multivendor BraTS dataset, see Menze et al [11]. Please note that the ground truth of BraTS 2015 was first produced by algorithms and then verified by annotators; in contrast, the ground truth of BraTS 2013 fused multiple manual annotations

Several methodological shortcomings of the present meta-analysis should be considered. First, various studies were excluded for the quantitative synthesis, due to missing data. Besides, heterogeneity of all analyses was considerably high, probably caused by technical variances of different MLA methodologies for segmentation. Lastly, only four out of 42 studies performed an out-of-sample external validation, emphasizing the importance of external validation to assess the robustness. It is probable that publication bias was present as there is no interest in the publication of poorly performing MLAs. In addition, differences in MR sequence input, ground truth, and other variables could play a role with regard to the outcomes, although this was considered a minor limitation as the source data across studies was similar in most studies.

Future gains of research on this topic may include an ensemble approach, as this might significantly boost the performance of segmentation. Thus, in addition, to focus current research on training individual segmentation systems, it may be interesting to investigate the fusion of multiple systems as well (i.e., segmentation of different imaging features in order to obtain different imaging biomarkers) [11]. Lastly, all included studies used retrospectively collected data, most of which using data from the BRATS databases. In order to further validate the performance of segmentation systems in clinical practice, larger-scale and external validated studies are preferred. In addition, data availability and providing online tools or downloadable scripts of the used MLAs could enhance future developments within this field of research significantly.

## Conclusion

In this study, a systematic review and meta-analysis of different studies using MLA for glioma segmentation shows good performance. However, external validation is often not carried out, which should be regarded as a significant limitation in this field of research. Therefore, further verification of the accuracy of these models is recommended. It is crucial that quality guidelines are followed when reporting on MLAs, which includes validation on an external test set.

## Supplementary Information

ESM 1 (DOCX 19 kb)

## Abbreviations

AI: Artificial intelligence
BraTS: Brain tumor segmentation
CI: Confidence interval
DSC: Dice similarity coefficient
GBM: Glioblastoma multiforme
HGG: High-grade glioma
LGG: Low-grade glioma
MLA: Machine learning algorithm
MRI: Magnetic resonance imaging
SD: Standard deviation
SE: Standard error

## Declarations

### Guarantor

The scientific guarantor of this publication is professor Mathias Prokop (MD, PhD) of Radboudumc, Nijmegen, The Netherlands.

### Statistics and biometry

Biostatistician Dr. Rogier Donders kindly provided statistical advice for this manuscript. Also, multiple authors have significant statistical expertise.

### Study subjects or cohorts overlap

All of the included studies have been previously reported, either as an original research paper or a conference paper.

### Methodology

• Systematic review

• meta-analysis
